# Direct evidence of multichannel-improved charge-carrier mechanism for enhanced photocatalytic H_2_ evolution

**DOI:** 10.1038/s41598-017-12203-y

**Published:** 2017-11-23

**Authors:** Jiangtao Zhao, Peng Zhang, Zhuo Wang, Shijie Zhang, Hongqing Gao, Junhua Hu, Guosheng Shao

**Affiliations:** 1 School of Materials Science and Engineering, Zhengzhou University, Zhengzhou, 450001 People’s Republic of China; 2 State Centre for International Cooperation on Designer Low-carbon and Environmental Materials (SCICDLCEM), Zhengzhou University, Zhengzhou, 450001 Henan People’s Republic of China; 3 Institute for Renewable Energy and Environmental Technologies, University of Bolton, Bolton, BL35AB UK

## Abstract

In the field of photocatalysis, the high-charge recombination rate has been the big challenge to photocatalytic conversion efficiency. Here we demonstrate the direct evidence of multichannel-improved charge-carrier mechanism to facilitate electron-hole transfer for raising photocatalytic H_2_ evolution activity. Scanning electron microscopy (SEM), transmission electron microscopy (TEM), X-ray photoelectron spectroscopy (XPS), X-ray diffraction (XRD), and UV-Vis diffuse reflectance spectroscopy (DRS), were used to characterize the as-fabricated samples. The result shows that the present design of Au/Pt nanoparticles (NPs) decorated one-dimensional Z-scheme TiO_2_/WO_3_ heterostructure composite nanofibers have been fabricated, which even exhibited excellent light absorption in the visible region and greatly enhanced photocatalytic activities on H_2_ generation comparing with pure TiO_2_, TiO_2_/WO_3_ and Pt/WO_3_/TiO_2_ nanofibers. This greatpromotion is mainly on account of the photosynthetic heterojunction system, which include the surface plasmon resonance (SPR) of Au nanoparticles, low overpotential of Pt nanoparticles, and more importantly, the one-dimensional multichannel-improved charge-carrier photosynthetic heterojunction system with Pt as an electron collector and WO_3_ as a hole collector. Transferring photoinduced electrons and holes at the same time, leading to effective charge separation was directly proved by ultraviolet photoelectron spectroscopy, electrochemical impedance spectroscopy, photocurrent analysis and incident photon-to-electron conversion spectrum.

## Introduction

The hydrogen energy has been a possiblel candidate for clean and sustainable power source, which provide a valuable way to settle the increasingly urgent energy crisis^1–4^. Over the past dacades, titania (TiO_2_) has been widely used in many fields of environmental protection, largely due to its strong chemical stability,thermal stability, non-toxic and low cost^5–7^. However, there are several shortcomings have limited the application of TiO_2_: i) the band gap of TiO_2_ is large (Eg = 3.2 eV), so it only absorbs the ultraviolet region which is about 3% of the overall solar intensity^3,8,9^. ii) large overpotential for hydrogen production^10^. iii) low quantum yield of TiO_2_ due to the rapid recombination of photogenerated charge carriers^11^.

Researchers have made much attempts to facilitate the utilization of TiO_2_ aiming at its poor visible light absorption, which increased its absorption in the visible region. Recent studies have recognized, decorated with noble metals^12–15^, such as gold (Au) and silver (Ag), will accelerate the visible light absorption because the surface plasmon resonance (SPR) root in the collective coherent oscillation of surface electrons^16,17^. Thereby, the plasmonic metal has been a potential candidate for extending the photoresponse range of wide bandgap semiconductors^18,19^. For instance, the Au NPs of SPR can enhance the visible-light absorption in Au/TiO_2_ nanostructure, which in return enhance the photocatalytic activity of TiO_2_^20–22^. However, the photocatalytic activities only relying on SPR can not meet our requests because of the large overpotencial of plasmonic metal. With the purpose of high efficiency of H_2_ evolution, it is desirable to modify Pt NPs into composite structures would be by combining the superiority of plasmonic metal and activation effect of Pt NPs for hydrogen production. Pt possesses the special advantage because of its small work function and low overpotential for energy conversion^23^, but lack visible-light absorption^24,25^. Therefore, in our studies, we load Pt and Au NPs into semiconductors, which exhibit synergy effect to improve the photocatalytic performance.

Nevertheless, the photocatalytic activity of TiO_2_ with noble metal could not meet our requests, which may be on account of short electron–hole pairs lifetime. The vast majority of studies focus on transferring photoinduced electrons but ignoring capturing the holes. Inspired by the basic structure of solar cell device and our previous work^26,27^, it is feasible to construct another route to capture or remove the photo-induced holes efficiently because it not only can capture photo-induced holes reducing the recombination of photoinduced electrons and holes, but also preserves outstanding redox ability. However, a large numbwe of the synthesized Z-scheme photocatalytic systems usually had redox pair (Fe^3+^/Fe^2+^, IO^3−^/I^−^) in solution, which would leading to some trouble in their practical applications^28,29^. Therefore, it is indispensable to design and construct solid-state Z-scheme photocatalytic system by introducing novel semiconductor with matchable bandgap and conduction band (CB) potential, such as TiO_2_/WO_3_, TiO_2_/ZnO, TiO_2_/SnO_2_, and TiO_2_/V_2_O_5_^30–32^. We have found^33^ that coupling WO_3_ with TiO_2_ can form an artificial solid-state Z-scheme^34–37^ heterterostructure system, accelerating the separation of electron-hole pairs by transferring photoelectrons from CB of WO_3_ to VB of TiO_2_. So that, the photoexcited electrons in CB of TiO_2_ would show strong reducibility and the photoexcited holes of valence band (VB) of WO_3_ would exhibit strong oxidizability, respectively^38–41^.

Herein, in this work, we reported a successful fabrication of an artificial multi-component photocatalytic system through facile electrospinning technique and followed calcinations treatment. The multichannel photosynthetic heterojunction system composed of TiO_2_, WO_3_, Pt and Au provides a significant way to settle the problem of low light-harvesting efficiency, low overpotential and low quantum yield of TiO_2._ The research of photocatalytic activity indicated that the as-fabricated nanocomposites presented greatly enhanced photocatalytic H_2_-evolution rates under simulated solar-light irradiation. Moreover, the special quaternary photosynthetic system with Pt and WO_3_ served as an electron collector and a hole collector based on Z-scheme has good suppression of electron-hole recombination, moreover, the increasing photocatalytic performance might be also due to the high photon absorption efficiency in visible region by Au NPs and WO_3_. The cocatalysts facilitated the separation and migration of photo-excited charges towards three different components thereby decreasing recombination as well as the reverse reaction, which was further confirmed by ultraviolet photoelectron spectroscopy (UPS), electrochemical impedance spectroscopy (EIS), photocurrent analysis and incident photon-to-electron conversion efficiency (IPCE). More importantly, it is advantageous about the unique structure composed of free-standing nanofibers, which could heighten not only charge carrier lifetime and transport rate, but also the light-scattering behavior^42–44^.

## Results and Discussions

### The structure and morphology of the composite nanofibers

**Figure 1:**
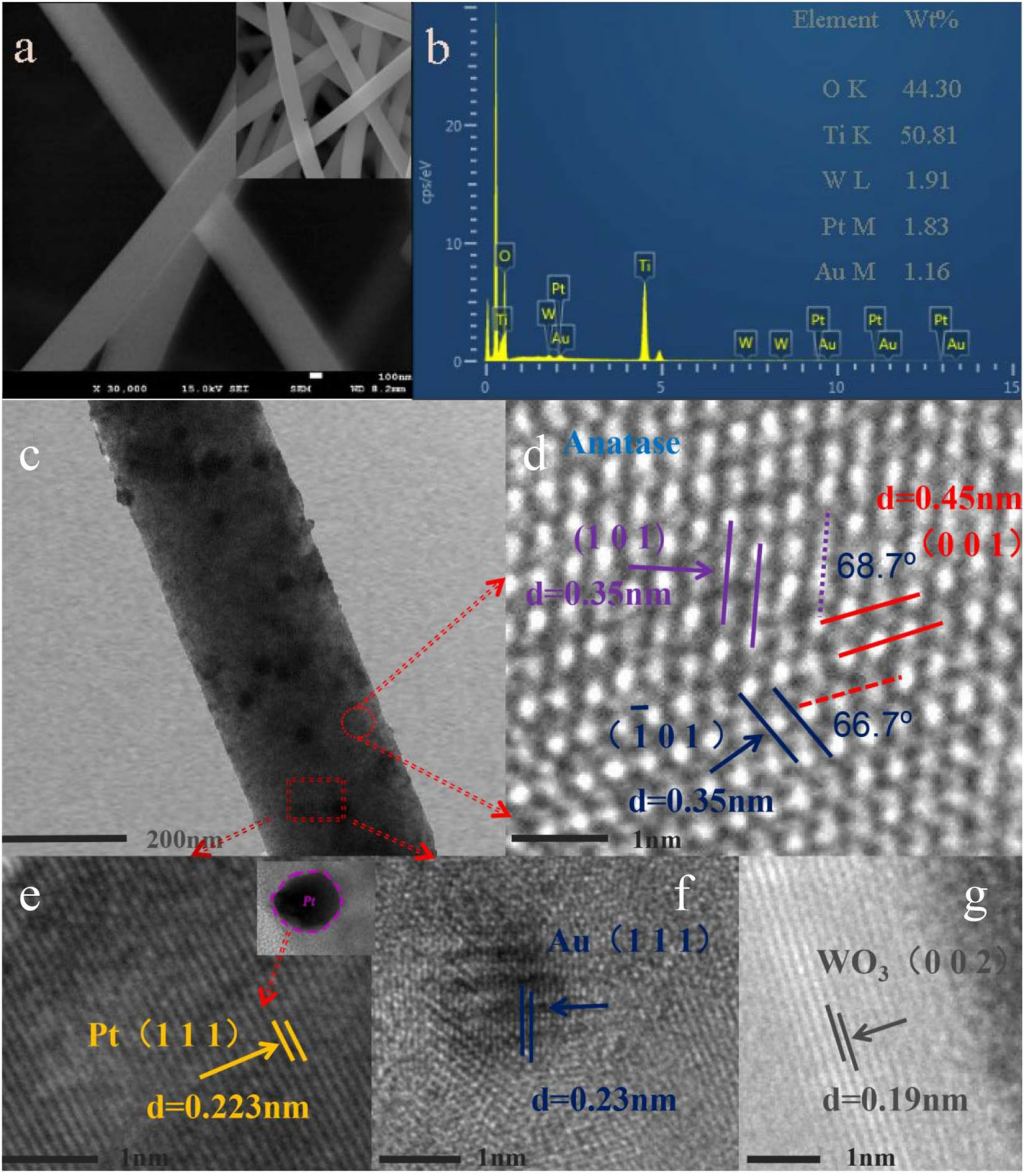
(**a**) SEM image of as-prepared Au/Pt/WO_3_/TiO_2_ NFs (inset: the shrunken SEM image); (**b**) EDX spectrum of the Au/Pt/WO_3_/TiO_2_ NFs; (**c**) TEM image of the Au/Pt/WO_3_/TiO_2_ NFs; (**d**–**g**) HRTEM images of the Au/Pt/WO_3_/TiO_2_ NFs.

Figure 1a showed the typical SEM image of Au/Pt/WO_3_/TiO_2_ NFs for representation (inset: the shrunken SEM image), which reveals that the as-electrospun samples have continuous fibrous structures with a diameter of ~300 nm. Both the images showed that the electrospun nanofibers aligned in random orientations and interweaved, and the high specific surface area of the NFs was confirmed by BET in Supplementary Table S1. Additionally, the EDX spectra of the Au/Pt/WO_3_/TiO_2_ NFs were applied to confirm the chemical components of the structure (Fig. 1b). The results demonstrated that Ti, O, W, Pt and Au elements existed in the Au/Pt/WO_3_/TiO_2_ NFs, which showed clearly that the content of W, Pt, Au and Ti was similar to the theoretical value, and it is also evidenced by XPS in Supplementary Table S2. For the sake of more details about the morphology structure and crystalline information of the one-dimensional Z-scheme Au/Pt/WO_3_/TiO_2_ heterostructures, the TEM and high-resolution transmission electron microscopy (HRTEM) observation were carried out. TEM image (Fig. 1c) indicated that the modified noble metal NPs with the uniform size and well-dispersed distribution could be readily distinguished from the NFs matrix because of the high electron density. As could be seen in Fig. 1c, it showed the typical TEM image of the one-dimensional Z-scheme Au/Pt/WO_3_/TiO_2_ composite nanofibers. At the same time, the high-resolution images of the Pt/Au/WO_3_/TiO_2_ nanofibers were shown in Fig. 1d–g. In Fig. 1d, the sample showed the characteristic spacings of 0.35 nm, 0.35 nm and 0.45 nm were consistent with the (1 0 1), (1 0 1) and (0 0 1) lattice plane of the TiO_2_, respectively. Figure 1e,f show the clear fringes spacing measured 0.223 nm and 0.23 nm, which corresponded to the (1 1 1) lattice spacing of Pt and (1 1 1) plane of Au. Besides, in Fig. 1g, the interplanar distances of 0.19 nm agreed well with the lattice spacing of the (0 0 2) plane of WO_3._ Therefore, the result suggests that the multi-component photosynthetic heterojunction system composed of TiO_2_, WO_3_, Pt and Au nanoparticles was well formed.

### X-ray diffraction (XRD) patterns

**Figure 2:**
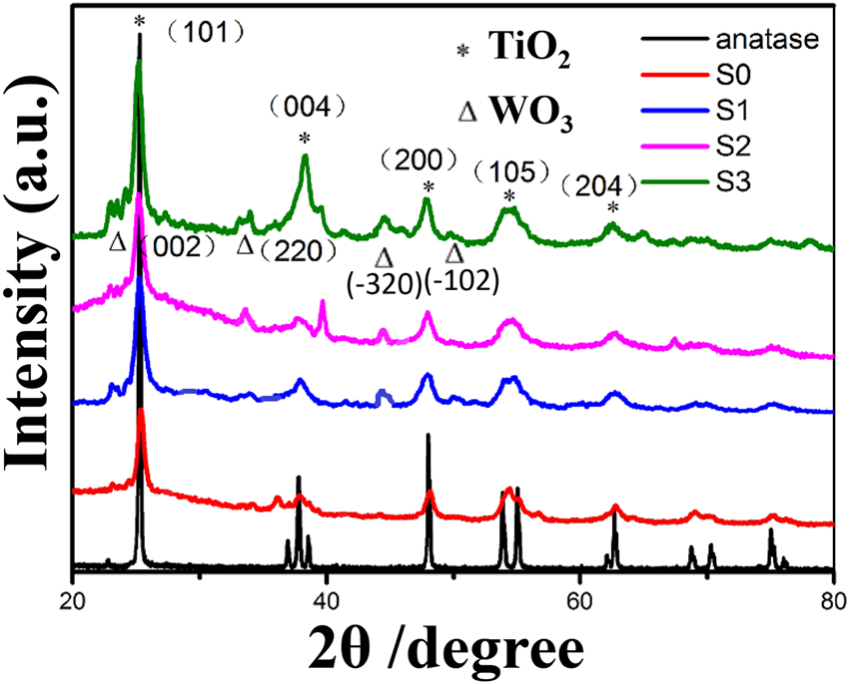
XRD patterns of the as-prepared samples and pure anatase.

X-ray diffraction (XRD) was conducted to assess the structure and phase purity of the Au/Pt/WO_3_/TiO_2_ NFs (S3). As a comparison, we also measured the pure TiO_2_ NFs (S0), the WO_3_/TiO_2_ NFs (S1) and the Pt/WO_3_/TiO_2_ NFs (S2). All the diffraction peaks in Fig. 2 could be perfectly indexed as the tetragonal anatase TiO_2_ (ICSD: 01-070-7348), whose peaks at 25.3°, 38.5°, 48.4°, 53.1° and 62.7° corresponded to (101), (004), (200), (105) and (204) planes of the anatase TiO_2_^45^. The diffraction peaks in S1, S2, S3 also could be perfectly indexed as the tungsten oxide’s (002) and (220) crystal planes, indicating that the well-crystallized TiO_2_/WO_3_ heterostructure in the as-electrospun nanofibers, which also evidenced in Supplementary Figure S1. The signal of Pt and Au nanocrystals was hardly to observable due to the very low concentration and minisize of Pt and Au in the sample. But through our EDX results, the content of the Pt and Au is 1.83 wt% and 1.16 wt%, respectively.

### XPS spectra

**Figure 3:**
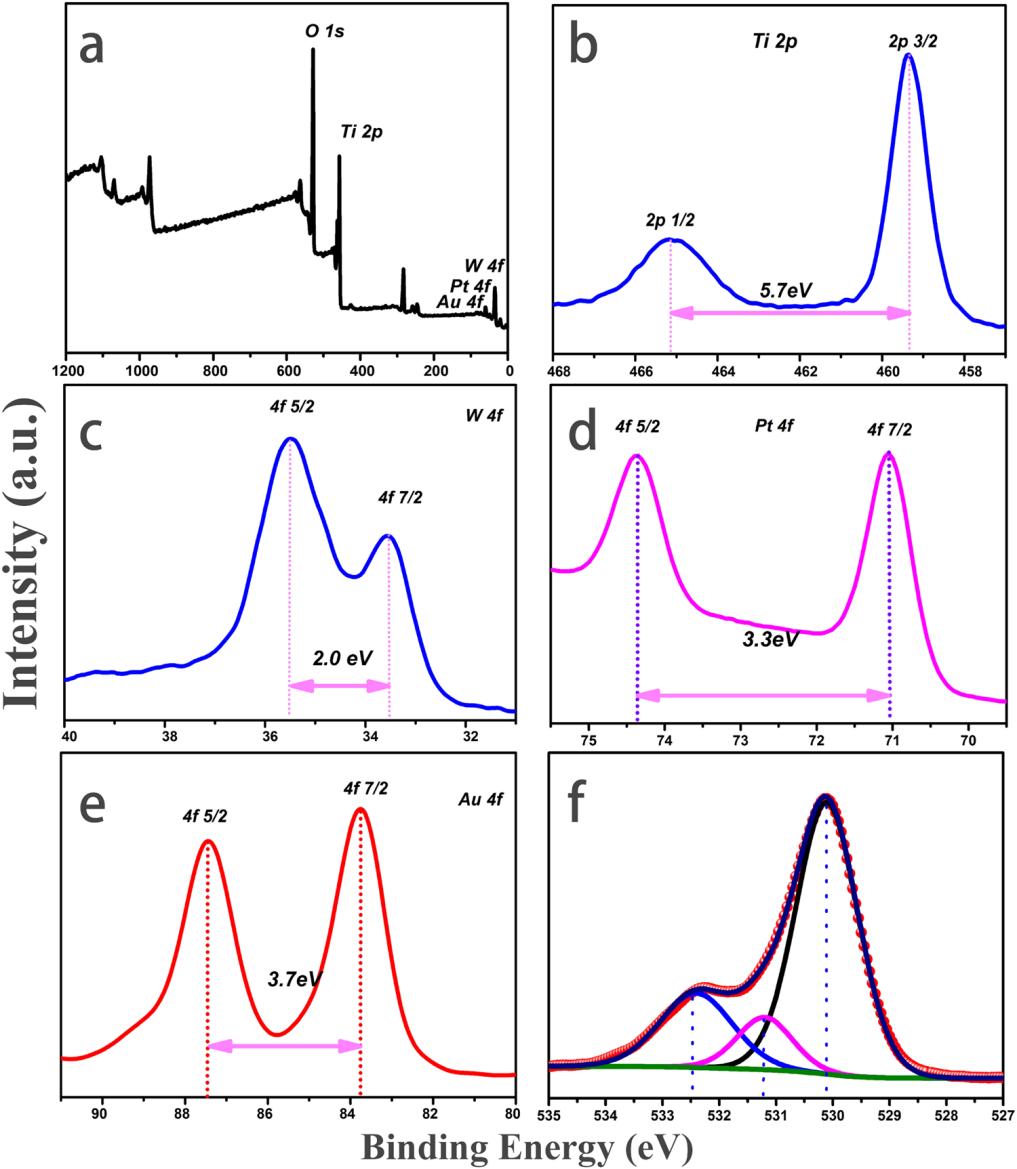
XPS spectra of the as-fabricated one-dimensional Z-scheme Au/Pt/WO_3_/TiO_2_ heterostructures: (**a**) fully scanned spectra; (**b**) XPS spectra of Ti 2p; (**c**) XPS spectra of W 4f; (**d**) XPS spectra of Pt 4f; (**e**) XPS spectra of Au 4f; (**f**) XPS spectra of O 1 s.

In order to confirm the chemical composition and various elements of the as-prepared samples, and supplement the result of XRD patterns, X-ray photoelectron spectroscopy (XPS) was carried out. The corresponding results are shown in Fig. 3a–f. As shown in Fig. 3a, the fully scanned spectra indicated that Ti, O, W, Pt and Au elements existed in as-fabricated products. Figure 3b shows XPS spectra of Ti 2p, and there were two peaks in the Ti 2p region. The peak located at 464.9 eV corresponded to the Ti 2p_1/2_ and another located at 459.2 eV was assigned to Ti 2p_3/2_. The splitting between Ti 2p_1/2_ and Ti 2p_3/2_ was 5.7 eV, indicating a normal state of Ti^4+^ in the as-prepared Pt/Au/WO_3_/TiO_2_ composite NFs. Figure 3c depicted the spin-orbit splitting of W 4 f and can be deconvoluted into a doublet with binding energy peaks at 35.5 eV and 37.5 eV, resulting from the emission of W 4f_7/2_ and W 4f_5/2_ core-levels that might belong to the W^6+^ oxidation state of tungsten atoms, which was in good agreement with previously reported results. As observed in Fig. 3d, the peak centered at 71.1 eV corresponded to the Pt 4f_7/2_ and another centered at 74.4 eV was assigned to Pt 4 f_5/2_, indicating a normal state of Pt° in the Pt/Au/WO_3_/TiO_2_ NFs. The Au 4f signals are shown in Fig. 3e. The presented Au 4f_7/2_ and Au 4f_5/2_ peaks are located at 83.8 eV and 87.5 eV, which corresponded to the values of the metallic Au° state^46^ and well supplement the XRD data in Fig. 2. Figure 3f presents the spectra of O 1 s for the Pt/Au/WO_3_/TiO_2_ NFs. According to the binding energy, the O 1 s spectrum make clear that there might be more than one chemical state, where peaks at 530.1 eV, 531.2 eV and 532.5 eV related to Ti–O (lattice O), W-O (lattice O) and surface hydroxyl groups (O-H), respectively. All of these XPS results gave the insight of the chemical bonding information of the multichannel-improved charge-carrier Au/Pt/WO3/TiO2 heterostructures.

### Characterization of the electronic structure of the composite catalyst

**Figure 4:**
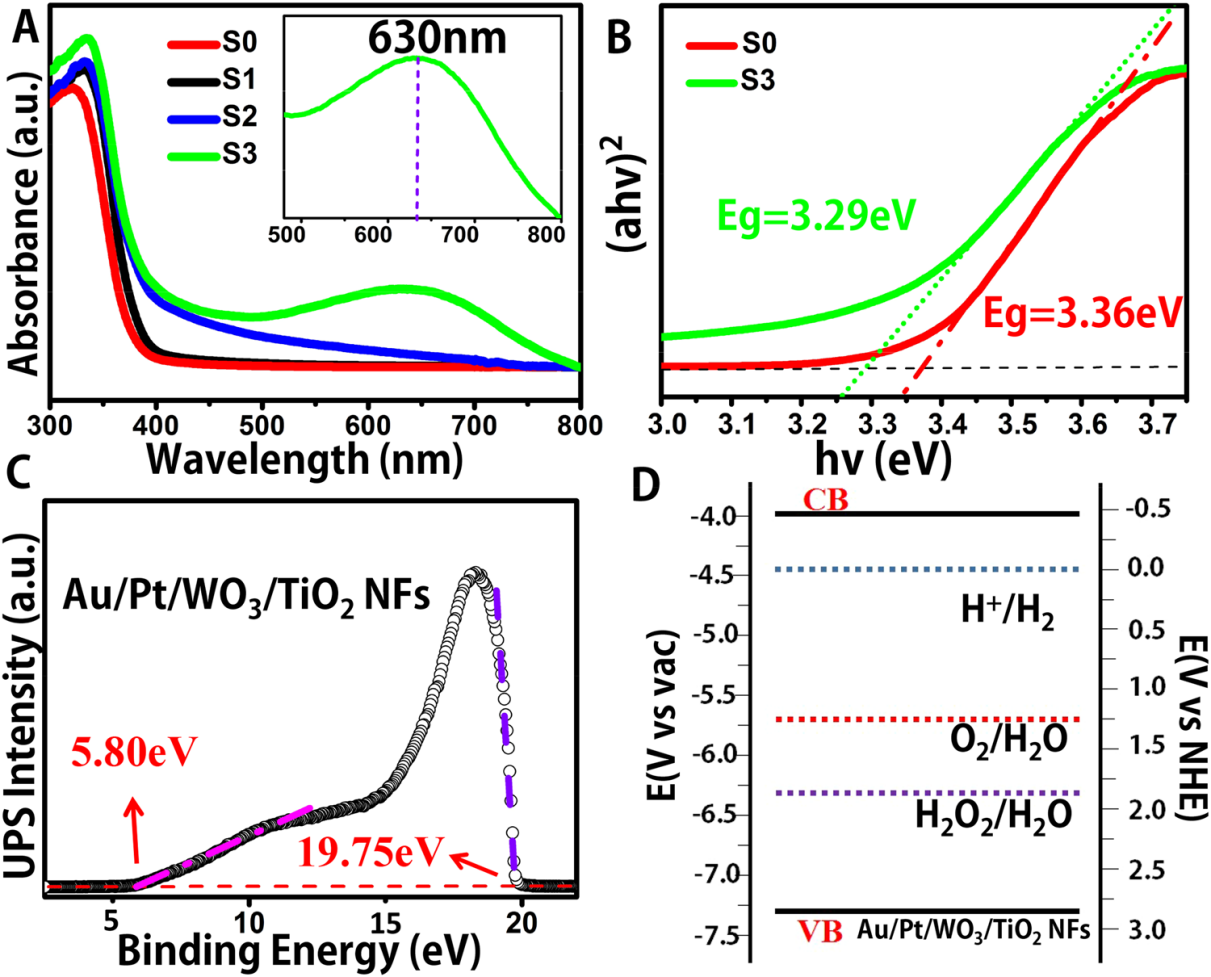
(**A**) UV-vis diffuses reflectance spectra of the as-fabricated NFs; (**B**) (*ahv*)^2^ versus *hv* curve of Au/Pt/WO_3_/TiO_2_ NFs (green curve) and TiO_2_ NFs (red curve); (**C**) UPS spectra of Au/Pt/WO_3_/TiO_2_ NFs (black dots); (**D**) Band structure diagram for Au/Pt/WO_3_/TiO_2_ NFs.

Figure 4A shows the UV−vis absorption spectra of the as-electrospun NFs, which are converted from the measured diffuse reflectance spectra by means of the Kubelka−Munk Function. The intense UV absorption band below 400 nm could be corresponded to the intrinsic bandgap absorption of anatase TiO_2_ (Eg: 3.2 eV). Compared with pure TiO_2_ NFs, there was a certain redshift after being composited with WO_3_, as WO_3_ acts as a hole collector, which prompting the separation of photoinduced electron-hole pairs. In terms of Pt/WO_3_/TiO_2_ nanofibers, the SPR absorption peak is hardly observed because of the high imaginary part of the dielectric function of Pt^47^. Although the addition of Pt could not alter substantially the absorption ability of nanofibers, a tiny increase of absorption in the visible region was observed. In this work, our as-fabricated one-dimensional Z-scheme Au/Pt/WO_3_/TiO_2_ heterostructure composite NFs showed obvious improvement in the visible range with a broad peak centered at around 630 nm which can be attributed to the SPR of embedded Au NPs, and it also was confirmed by fluorescence spectrum shown in Supplementary Figure S2. Furthermore, it is necessary to evaluate the bandgap of the samples, thus we applied UV-vis absorption spectrum for calculating the bandgags of semiconductors, referring to previous work claiming TiO_2_ to be a direct band gap material^48^. The Eg value of Au/Pt/WO_3_/TiO_2_ heterostructure NFs was calculated to be 3.29 eV (Fig. 4B, green curve), which is compared with that of pure TiO_2_ NFs (Figs 4B, 3.36 eV, red curve). The Tauc plot curve of Au/Pt/WO_3_/TiO_2_ NFs also indicated that it is useful for enhancing the light absorbance and the photocatalytic ability.

Furthermore, in order to confirm the ionization potential of as-prepared Au/Pt/WO_3_/TiO_2_ NFs, which played a guiding function over the water splitting, we used ultraviolet photoelectron spectroscopy (UPS) for characterizing the as-prepared Au/Pt/WO_3_/TiO_2_ NFs, and it was calculated to be 7.27 eV by subtracting the width of the He I UPS spectra (Fig. 4C) from the excitation energy (21.22 eV). The Ec (conduction band energy) is thus calculated at 3.98 eV from Ev – Eg. In addition, We illustrate the effective energy band structure about the Eg, Ev, and Ec values about Au/Pt/WO_3_/TiO_2_ NFs in Fig. 4D, from which we also obtained the energy potentials according to RHE. These bands are suitably positioned to permit transfer of electrons and holes for water splitting, therefore corroborating the unique structure of Au/Pt/WO_3_/TiO_2_ NFs as a photocatalyst for overall water splitting.

### Photoelectrochemical test

**Figure 5:**
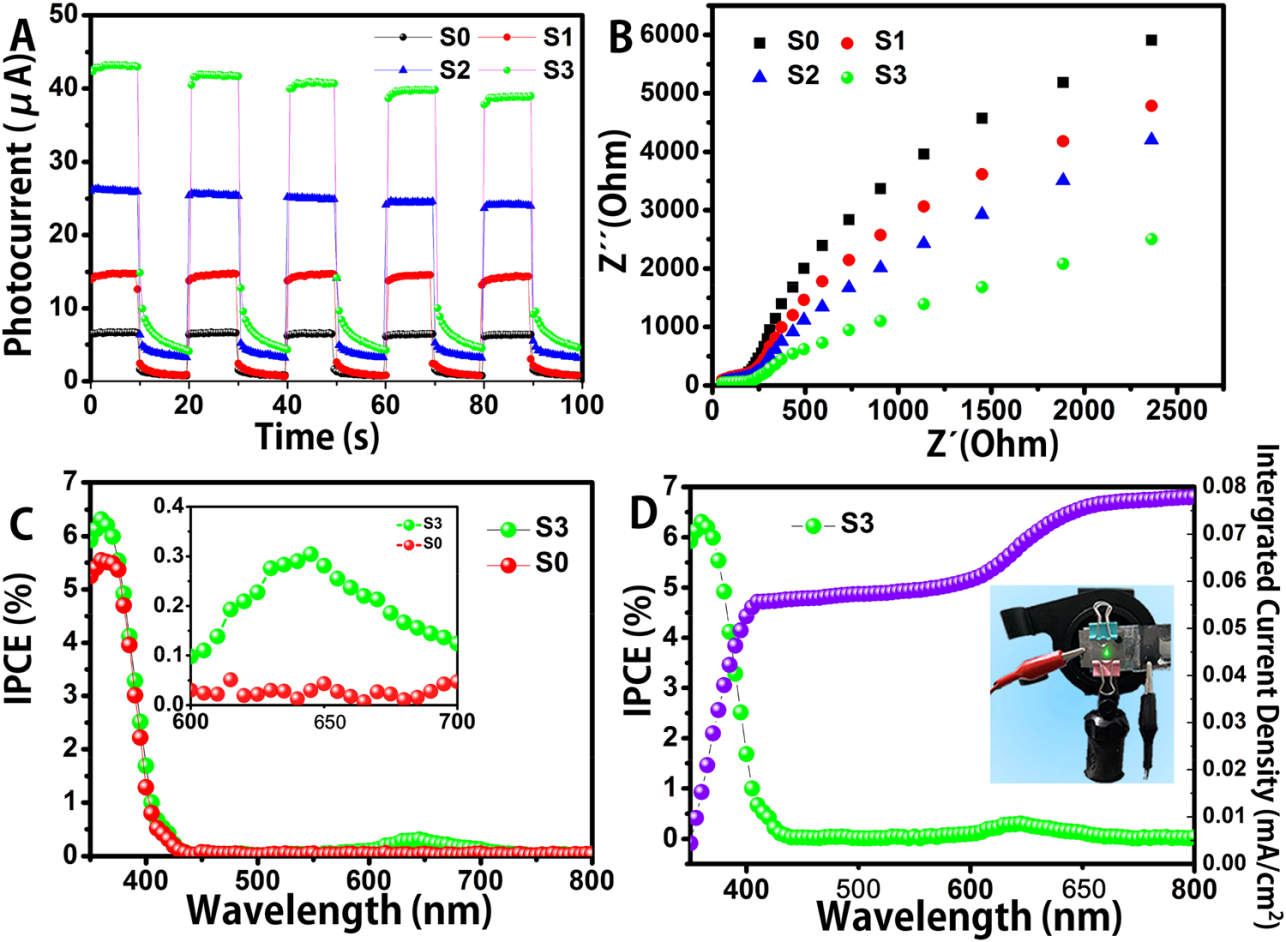
(**A**) Photocurrents of the as-fabricated NFs electrodes under full spectrum light ittadiation; (**B**) EIS Nyquist plots of the nanofiber electrodes; (**C**) IPCE spectrum of the samples; (**D**) the corresponding IPCE spectrum and intergrated current density.

The photocatalytic performances of as-prepared composite NFs were appraised by testing the photocurrents and the electrochemical impedance spectroscopy (EIS). The recombination rate of photoinduced electron-hole pairs can be evaluated by detecting the photocurrent. It is effective to qualitatively study the excitation and transfer of photogenerated charge carriers by photoelectrochemical measurements. Photocurrents were measured with 0.2 M Na_2_SO_4_ solution as electrolyte and gives an apparent response to light on/off for TiO_2_ NFs, WO_3_/TiO_2_ NFs, Pt/WO_3_/TiO_2_ NFs and one-dimensional Z-scheme Au/Pt/WO_3_/TiO_2_ NFs electrodes under full spectrum light (Fig. 5A). It was clear that fast and uniform photocurrent responses were observed in all electrodes and the photoresponsive phenomenon was reversible. Under simulated sunlight irradiation, the photocurrent of the Au/Pt/WO_3_/TiO_2_ NFs electrode was about 6 times as high as that of the TiO_2_ NFs, about 3 times higher than that of WO_3_/TiO_2_ NFs, and about two times as high as that of Pt/WO_3_/TiO_2_ NFs (Fig. 5A), indicating that our design idea is feasible. Such obviously differences between these photoelectrodes probably owing to the enhanced light absorption, efficient electron-hole transfer and charge separation in the one-dimensional Z-scheme Au/Pt/WO_3_/TiO_2_ composite NFs.

The electrochemical impedance spectroscopy (EIS) measurement was employed to obtain more detailed information about the charge transport within our work. Figure 5B displayed the EIS of these four electrodes. All the Nyquist plots impedance spectra were in open-circuit potential conditions and showed similar. The nonlinear regression fitting using a conventional Randle’s circuit [R(QR)] routine gave active charge transfer resistance for: Au/Pt/WO_3_/TiO_2_ NFs < Pt/WO_3_/TiO_2_ NFs < WO_3_/TiO_2_ NFs < TiO_2_ NFs, indicating a markedly high hydrogen evolution reaction (HRE) catalytic activity for the one-dimensional Z-scheme Au/Pt/WO_3_/TiO_2_ heterostructure composite nanofibers, which indicated our prepared heterostructures composite nanofibers could reduce the charge-transfer resistance and enhance the electrolyte penetration. In our work, under the irradiation of solar-light, the photogenerated electrons of WO_3_ can be transferred to th**e** VB of TiO_2_, and recombined with the photogenerated holes of TiO_2_ in the VB, promoting an effective charge carries separation between WO_3_’s VB and TiO_2_’s CB. Moreover, the electrons of TiO_2_ can be transferred from the CB of TiO_2_ to Pt nanoparticles, further promoting charge separation, in addition, Au NPs could be excited under visible-light to high-energy level and inject into the conduction band (CB) of TiO_2,_ thus accelerating the photocatalytic process. These results suggested that both WO_3_, Pt and Au nanoparticles played important roles in improving the charge separation efficiency.

The results of both photocurrent and EIS indicated that the structure of optimal design by us exhibit increasing photoinduced electrons and holes separation, which could be on account of the enhanced electrical conductivity. Hereof, in order to get more details about quantum efficiency of our well-designed structure, we take the comparison of incident photo-to-current efficiency (IPCE) of pure TiO_2_ NFs and our designed composite NFs. It can be seen from Fig. 5C that the higher IPCE values of Au/Pt/WO_3_/TiO_2_ NFs electrodes over a wide range both in UV and visible light region was attributed to the increase in light capture and conversion ability. As will be described below, the distribution of Au NPs also plays an important role in the structure. Figure 5D shows the IPCE and the intergrated current density of the sample S3, the intergrated current calculated from the IPCE matches well with the photocurrent to some extent, and the tiny deviation may be caused by the different design of the device. Therefore, it is obvious that the structure we designed revealed higher quantum efficiency under the light irradiation.

### Photocatalytic hydrogen production activity and the simulation of the distribution of Au NPs

**Figure 6:**
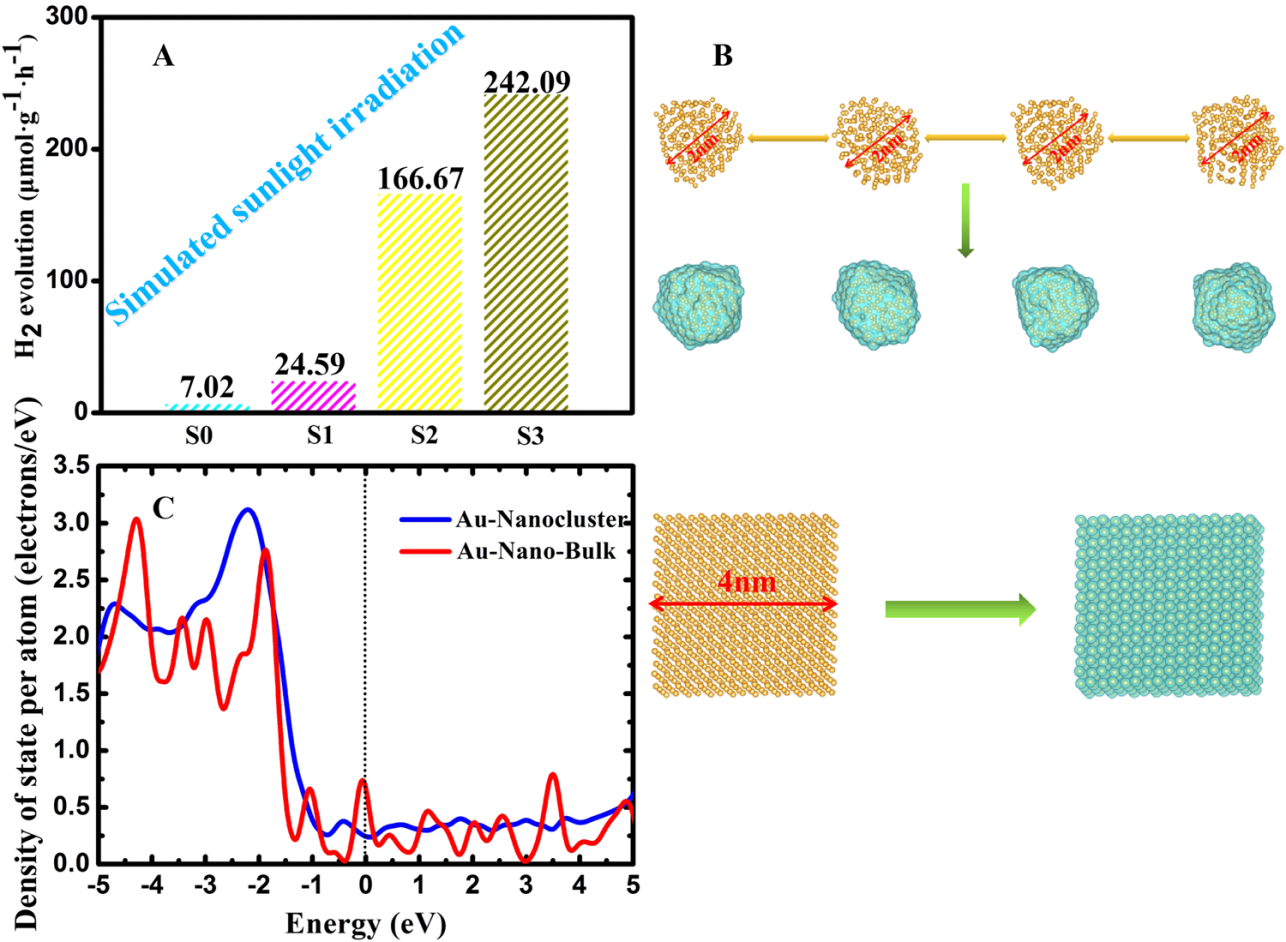
(**A**) H_2_ production rates of the different samples; (**B**,**C**): Electronic state and DOS of the Au nanocluster and Au nano-bulk.

The photocatalytic hydrogen evolution over different samples was evaluated under Xe arc lamp irradiation. Control experiments in the absence of TiO_2_ NFs, WO_3_/TiO_2_ NFs and Pt/WO_3_/TiO_2_ NFs showed different amount of H_2_ production. As is shown in Fig. 6A, there was a very low H_2_ production rate in TiO_2_ NFs which is almost undetectable because of the fast recombination of photogenerated charge carriers of TiO_2_. Relatively, the one-dimensional Z-scheme WO_3_/TiO_2_ NFs showed much higher H_2_ production rate (24.59 μmol·g^−1^ h^−1^) due to the WO_3_ in NFs could act as a hole collector and control the charge recombination process. After Pt-decorated WO_3_/TiO_2_ NFs, the production of H_2_ has a more increasing because Pt NPs can work as an electron-sink and active reaction sites for H_2_ production. Notably, H_2_-production of the one-dimensional Au/Pt/WO_3_/TiO_2_ heterostructure composite NFs has significantly improved compared with pure TiO_2_ nanofibers, TiO_2_/WO_3_ nanofibers, and Pt/WO_3_/TiO_2_ nanofibers, reached to 242.09 μmol·g^−1^h^−1^. Moreover, in order to better illustrate the SPR effect of Au, we prepared Au/TiO_2_ NFs, and studied its photocatalytic performance in Supplementary Figure S3, the hydrogen production rate is shown in Supplementary Figure S4. In addition, the cycling performance and stability of photocatalysts was discussed in Supplementary Figure S4, Supplementary Figure S5 and Supplementary Figure S6. It is noteworthy that the addition of Au NPs had important meaning on photocatalytic activity, to have a better understanding how the properties of the NFs are affected by the dispersion of Au NPs and for comparison with the other structural models, we simulate two structures with different size of the distribution of Au NPs. After whole relaxation process finished, the nanoparticle Au with diameter of about 2 nm had been obtained as presented in Fig. 6B. At same time, abundant charge density can be observed distributing on the surface of nanoparticle, while it is uniform distributing around each Au atom in the bulk model. Moreover, for the density of electron state (DOS) as displayed in Fig. 6C, the DOS of Au nanoparticle is much more continuous than Au bulk in the region of Fermi lever. It suggests that electrons could be much easier transfer from Au nanoparticle to (other materials) with the assistant of these uninterrupted electron levels.

### Postulated photocatalytic mechanism

As discussed above, the absorption ability and efficient charge separation were important factors of the significant improvement of the photocatalytic ability. Based on the photocurrent and EIS results, an increasing photoinduced electron–hole separation and transfer exhibited in the composites NFs. As we all know, it was effevtive to confirm the increased photocatalytic performance by the incident photon-to-electron conversion (IPCE) spectrum. Figure 5C showed clearly that the Au/Pt/WO_3_/TiO_2_ sample markedly enahnced the photoresponse in the UV-visible light region compared to the TiO_2_, which is corresponded to the absorption data. Moreover, we simulate two examples with different size of Au NPs, and the electronic states indicated the dispersive Au NPs play an important role in the composite, otherwise, the composite(compared to Pt/WO_3_/TiO_2_) for hydrogen production in response to visible light irradiation might be attributed to the near-field electromagnetic effect induced by SPR of Au nanoparticles.

**Figure 7:**
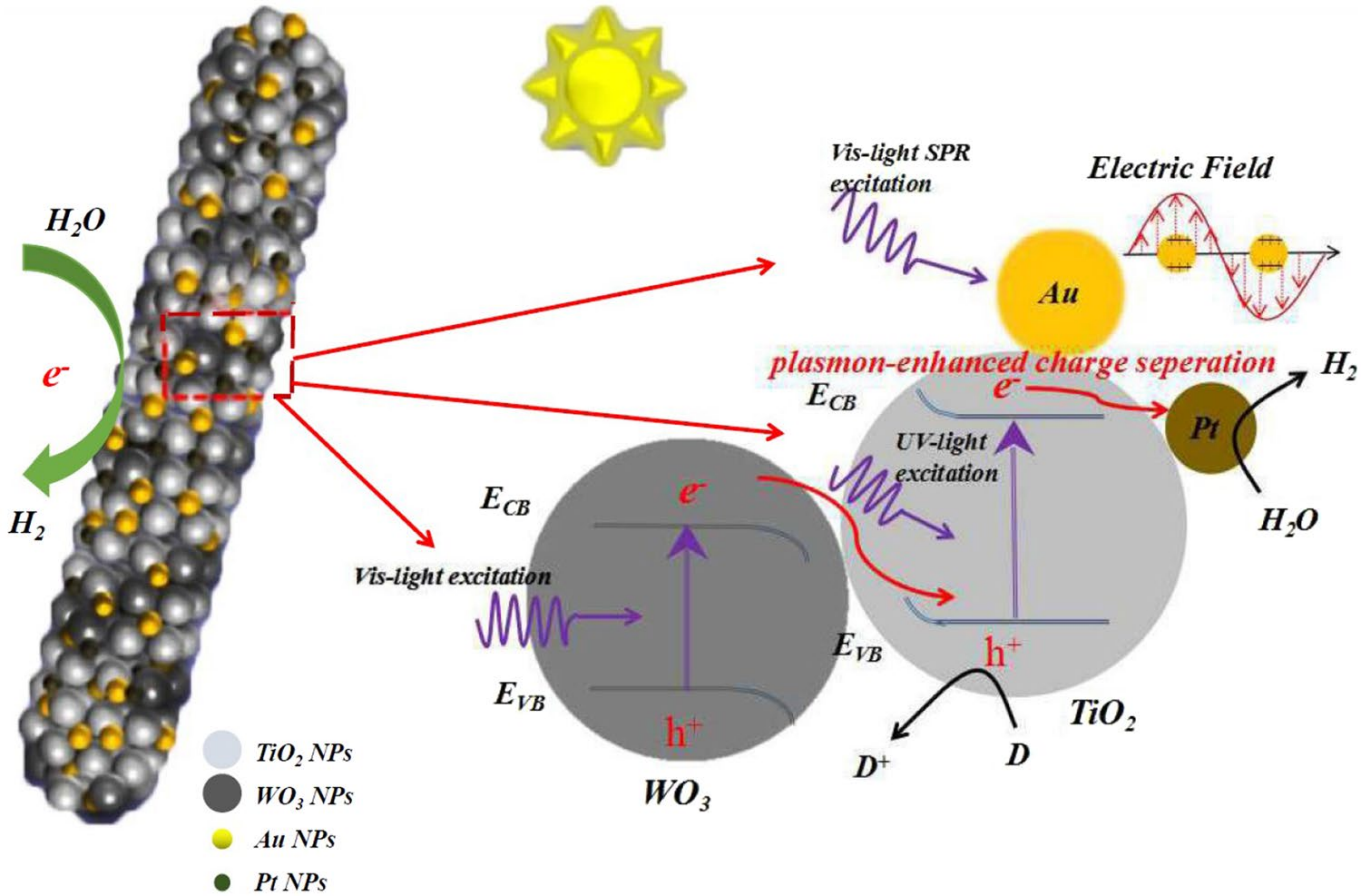
Schematic diagram showing the photocatalytic for H_2_ production on the Au/Pt/WO_3_/TiO_2_ heterostructure composite NFs.

On the basis of the above results and discussion, a plausible mechanism and a schematic drawing of the electron–hole separation process were proposed to explain the enhanced photocatalytic activity of the Au/Pt/WO_3_/TiO_2_ composite NFs. As illustrated in Fig. 7, the Au/Pt/WO_3_/TiO_2_ composite follows a typical Z-scheme transfer system rather than a traditional intraband transfer system. Under ultraviolet light irradiation, both TiO_2_ and WO_3_ could be excited, and the photogenerated electrons in the CB of TiO_2_ can easily shift into the Pt nanoparticles through the Schottky barrier because of its small work function and low overpotential of Pt, left the holes on the VB of TiO_2_. Meanwhile, the electrons in the CB of WO_3_ will transfer into the VB of TiO_2_, avoiding the recombination of electrons in TiO_2_’s CB and holes in WO_3_’s VB. In this interband transfer way, the photogenerated electrons and holes can be efficiently separated, which further leads to increased photocatalytic ability. On the other hand, according to the SPR effect of Au NPs, it was contributed to the photocatalytic process through direct transfer of plasmon-excited “hot” electrons and/or the strongly enhanced local electric field under visible light irradiation. Upon SPR excitation, the surface electrons on Au NPs might be excited to high-energy level and inject into the conduction band (CB) of TiO_2_ through the Au-TiO_2_ interfaces for reduction reaction on TiO_2_ surfaces, thus promoting the photocatalytic process.

## Conclusions

In summary, we describe herein an effective route to synthesize the one-dimensional Z-scheme Au/Pt/WO_3_/TiO_2_ heterostructure composite nanofibers by combining the electrospinning and calcination method, with multichannel electrons transport mechanism to enhance the photocatalytic H_2_ production. Notably, our study demonstrates that this design of multi-component photosynthetic heterojunction system can harvest visible light for energy conversion, through Au SPR effect and a narrower bandgap WO_3_ composite. More important, the excited electrons in CB of TiO_2_ and injected electrons by Au nanoparticles are collected by Pt uniaxially, and the holes left in the VB which located at +2.15 eV (E vs. SCE) of WO_3_^49^. Therefore, it is expected that this one-dimensional photocatalytic with large surface area and multichannel electrons transport material system could improve the ability of hydrogen evolution and even greatly promote their commercial application in the field of clean energy.

## Methods

### Fabrication of Au/Pt/WO_3_/TiO_2_ Composite Nanofibers (NFs)

The Au/Pt/WO_3_/TiO_2_ NFs were fabricated by electrospinning and calcination. Typically, tetrabutyl titanate (Ti(OC_4_H_9_)_4_) (2.0 mL)(Aladdin, CP, 98.0%), 1.1 g of poly(vinyl pyrrolidone) (PVP) powder (Mw = 1 300 000) and a certain amount of HAuCl_4_ were added to a mixture solution containing 10 mL ethanol (Sinopharm Chemical Reagent, 99.7%) and 6 mL acetic acid (Sinopharm Chemical Reagent, 99.5%) under vigorous stirring. Then 0.15 g ammonium tungstate (Aldrich, 99.95%) (Our previous study showed a higher photocatalytic activity in this proportion) was slowly added into the solution under vigorous stirring. Subsequently, a certain amount of H_2_PtCl_6_·6H2O was added to this solution which was then kept vigorously stirring for 12 h at room temperature. Then the above precursor solutions were drawn into a hypodermic syringe for electrospinning. Finally, the above composite nanofibers were calcined in air at 520°C for 30 min with a heating rate of 3°C/min. Thus, the Au/Pt/WO_3_/TiO_2_ nanofibers (denoted as S3) were successfully prepared. As a comparison, we also prepared the TiO_2_ nanofibers(denoted as S0), TiO_2_/WO_3_ (denoted as S1) and Pt/WO_3_/TiO_2_ nanofibers (denoted as S2) using the same method.

### Characterization techniques

The morphologies and structures of the products were investigated by field emission scanning electron microscopy (FESEM; JSM-7500F) at 20 kV and transmission electron microscopy (TEM; FEI Tecnai G2 F20) with an accelerating voltage of 200 kV. Energy dispersive X-ray spectrum (EDX) was detected on EDAX TEAM. The X-ray photoelectron spectroscopy (XPS) and ultraviolet photoelectron spectroscopy (UPS) were measured by the multifunctional X-ray photoelectron spectroscopy (AXIS UltraDLD, Kratos Analytical Inc). The binding energy values were calibrated with respect to the C (1 s) peak (284.6 eV). The XRD (Rigaku Ultima IV) was carried out to determine the crystallization and the phase transition with Cu Ka radiation (wavelength = 0.15406 nm) from 20° to 80° at a scanning rate of 4°/min. The diffuse reflectance spectra of all samples were recorded on a UV-Vis spectrophotometer (Shimadzu, model UV 3600) equipped with an integrating sphere in the range of 300 to 800 nm and standard BaSO_4_ powder was used as a reference.

### Electrochemical measurements

Photoelectrochemical measurements were carried out in a quartz cubic urn with a conventional three-electrode process on an electrochemical workstation (AMETEK, PARSTAT 4000, America). The as-synthetic photoanode was the working electrode, and a Pt wire and Ag/AgCl electrode served as the counter electrode and reference electrode, respectively. The electrolyte was a 0.2 M Na_2_SO_4_ aqueous solution. The photoanode surface has an illuminated area of 1.5 × 1.5 cm^2^. All the samples (0.1 g) mixing with polyethylene glycol (0.05 g) and water (0.35 ml) were deposited on the FTO conducting glass, with thin transparent cover glass to seal avoiding samples fall off. The light source was a 300 W Xe lamp (Beijing Perfectlight Co. Ltd, PLS-SXE-300). The photocurrent response spectroscopy was carried out at a constant potential of +0.9 V to the working photoanode. Electrochemical impedance spectra (EIS) were measured at an open-circuit voltage. A sinusoidal ac perturbation of 5 mV was applied to the electrode over the frequency range of 100 mHz to 10 kHz. The IPCE of the samples was applied using a power source (Newport 300 W xenon lamp, 66920) with a monochromator (Newport Cornerstone 260) and a multimater (Keithley 2001). In our work, the IPCE spectrum was conducted as a function of wavelength from 350 to 800 nm by using the as-synthetic photoanode as working electrode and a Pt wire as the counter electrode (like the image inserted in Fig. 5D). All measurements were carried out at room temperature.

### Photocatalytic hydrogen generation

In a typical test, photocatalytic water splitting for hydrogen production was proceed on an equipment of online analysis system (LabSolar-III AG, Beijing Perfectlight Co. Ltd.) directly connected with gas chromatography (GC-7860). A 300 W Xe arc lamp (PLS-SXE 300, Beijing Perfectlight Co. Ltd) was used as the light source. The H_2_ evolution tests were detected in a quartz reactor. Typically, the as-electrospun NFs photocatalysts (50 mg) was added into the solution containing 40 mL aqueous solution and 25 mL methyl alcohol (anhydrous, Sinopharm Chemical Regent, 99.5%) under strong magnetic stirring for sufficient mixing. After vacuumizing, the reactor was exposed under a 300 W Xe lamp. The gas product composition from the upper space above the liquid suspension in the quartz reactor was periodically analyzed using a gas chromatography (GC-7860), equipped with a thermal conductivity detector (TCD). The advantage of online analysis system for hydrogen production is that we could test the amount of H_2_ by real-time monitoring and the generated gas was pumped to gas chromatography every 1 h to get the *in situ* average hydrogen production rate.

### Simulate two structures with different size of the distribution of Au NPs

The cluster structure build from 4 × 4 × 4 Au super-cell containing 256 Au atoms, while six vacuum layers with 20 Å thickness was added into the system along ±X, ±Y and ±Z axis to obtain Au cluster. The vacuum layers with large enough thickness completely cancelled the interaction from the neighbor unit. Theoretical calculations are performed using the Vienna *Ab initio* Simulation Package(VASP)^50,51^, with the ionic potentials including the effect of core electrons being described by the projector augmented wave (PAW) method^52,53^. In this work, the Perdew-Burke-Ernzerhof (PBE) GGA exchange-correlation (XC) functional^54,55^ are used to relax the structural configurations. For the geometric relaxation of the structures, summation over the Brillouin Zone (BZ) is performed with 1 × 1 × 1 Gamma point mesh. A plane-wave energy cutoff of 500 eV is used here. The whole geometric relaxation process can be divided into two processes.

Firstly, ab initio molecular dynamics (AIMD) simulation^54^ was used to melt the Au cluster assigned initial temperature of 1000 K for 5000 steps (10 ps), then gradually annealing down to the desired temperature (800 K, 600 K, 400 K) by velocity scaling over 1000 time steps(2 ps) for each annealing procedures. The time step of molecular dynamics is chosen to be 2fs for AIMD evolution with a NVT ensemble and a Nosé–Hoover thermostat. Secondly, the well-known conjugate gradient (CG) minimization method^55–59^ was adopted to further relax the Au cluster until the total force on each ion was reduced below 0.02 eV/Å.

## Electronic supplementary material

Direct evidence of multichannel-improved charge-carrier mechanism for enhanced photocatalytic H2 evolution
