# Supplementary material for: Direct evidence of multichannel-improved charge-carrier mechanism for enhanced photocatalytic H2 evolution
Source: Sci Rep. 2017 Nov 23;7:16116. doi: 10.1038/s41598-017-12203-y (PMC5701016; doi:10.1038/s41598-017-12203-y)
Supplement: Supplementary file 1 — Direct evidence of multichannel-improved charge-carrier mechanism for enhanced photocatalytic H2 evolution [file 41598_2017_12203_MOESM1_ESM.pdf]

Direct evidence of multichannel-improved charge-carrier mechanism for enhanced photocatalytic H_2 evolution

Jiangtao Zhao[1], Peng Zhang[1,2], Zhuo Wang[1], Shijie Zhang[1], Hongqing Gao[1], Junhua Hu[1,2] and Guosheng Shao[1,2,3]

[1]School of Materials Science and Engineering, Zhengzhou University, Zhengzhou 450001, People's Republic of China.

[2]State Centre for International Cooperation on Designer Low-carbon and Environmental Materials (SCICDLCEM), Zhengzhou University, Zhengzhou 450001, Henan, People's Republic of China.

[3]Institute for Renewable Energy and Environmental Technologies, University of Bolton, Bolton BL35AB, UK.

*Corresponding authors: State Centre for International Cooperation on Designer Low-carbon and Environmental Materials (SCICDLCEM), Zhengzhou University, 100 Kexue Street, Zhengzhou 450001, People's Republic of China.

E-mail: Zhangp@zzu.edu.cn； Hujh@zzu.edu.cn; Gshao@zzu.edu.cn

Supplementary Materials

Materials	BET Surface Area (m^2/g)
TiO_2 nanofibers	36.60
Au/Pt/WO_3/TiO_2 nanofibers	31.33

Supplementary Table S1. BET data of the materials

The nanofibers possess large specific surface area, which leads to a great number of active sites, and it in returns promotes the photocatalytic performance. The BET data was obtained by MicroActive for ASAP 2460 2.01.

Component	Mass conc. [%]	Error [%]
O 1s	43.89	0.33
Ti 2p	51.45	0.31
Au 4f	1.34	0.18
Pt 4f	1.68	0.11
W 4f	1.64	0.29

Supplementary Table S2. Experimental details and element contents determined by XPS

In order to determine the chemical components and element contents of the composite nanofibers, XPS was carried out, which is consistent with the result of EDX.

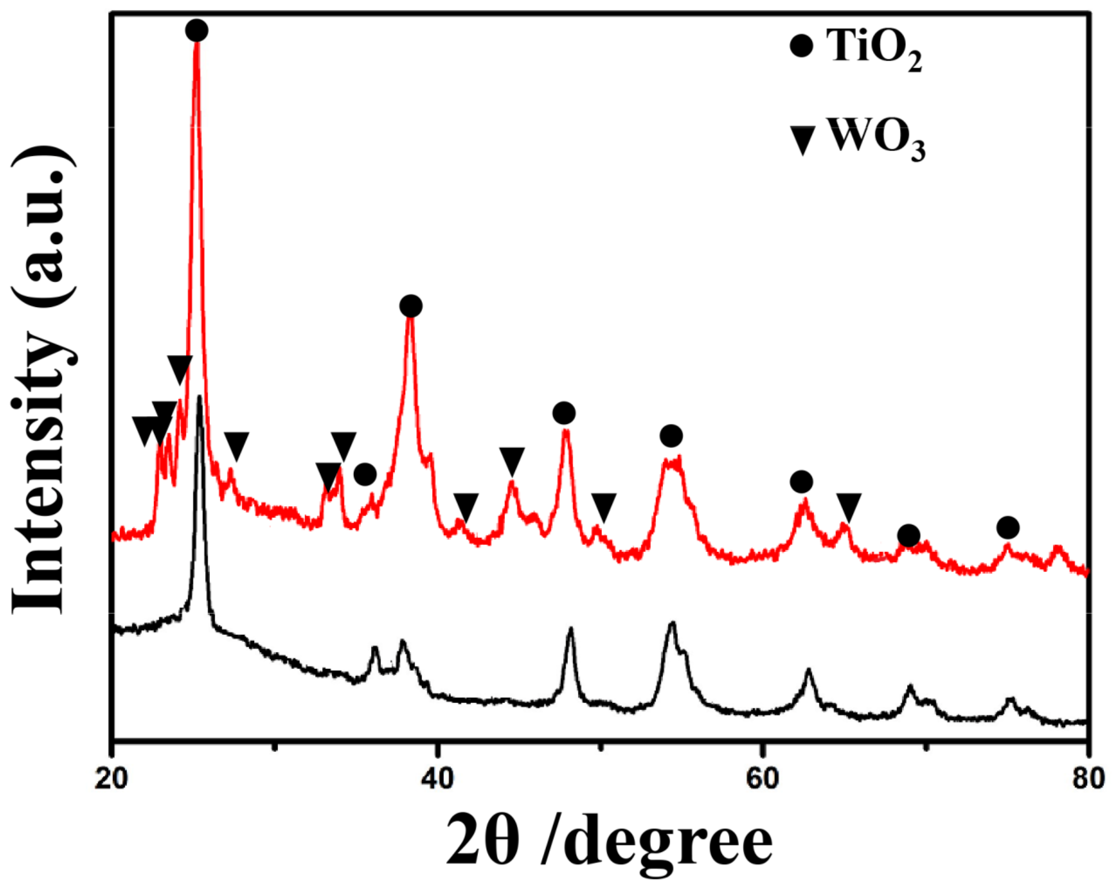

Supplementary Figure S1. XRD patterns of calcined TiO_2 and TiO_2/WO_3 NFs

On the purpose of confirming the purity of TiO_2 and WO_3 in the nanofibers, we repeat the characteration of calcined TiO_2/WO_3 nanofibers, and we mark all the peaks of TiO_2 and WO_3 in the patterns, As shown in the figure S1, the symbol of circle as corresponding to the anatase phase (JACPDS 21-1272), and the triangular symbol diffraction peaks at 23.27° ,23.77°, 24.43°, 26.78°, 33.99°, 34.35°, 41.90°, 44.47°, 49.28°, 65.23° are consistent with (0 0 1), (0 2 0), (2 0 0), (-1 2 0), (2 0 1), (2 2 0), (2 -2 1), (-3 2 0), (-1 0 2), (-5 0 1) of WO_3 (76-1734), respectively. It indicated the pure TiO_2/WO_3 phase in the nanofibers.

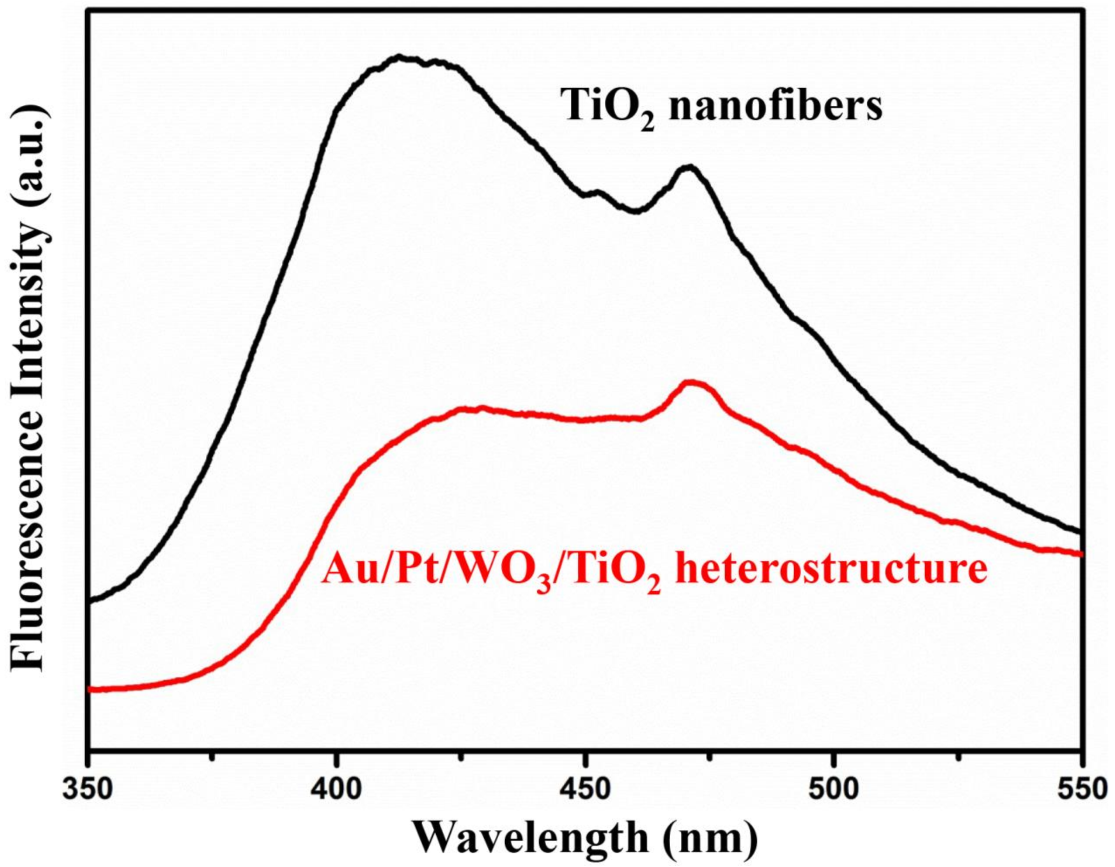

Supplementary Figure S2. Fluorescence emission spectrum of the samples

To further understand the formation of Z-scheme Au/Pt/WO_3/TiO_2 heterostructure, we applied the fluorescence spectrum for the TiO_2 nanofibers and Z-scheme Au/Pt/WO_3/TiO_2 composite heterostructure in Supplementary Figure S2. The lower fluorescence intensity indicated the lower recombination rate of photoinduced electron-hole pairs. The result demonstrated the Z-scheme heterostructure was contributed to suppressing the recombination of electrons and holes thus enhanced the photocatalytic ability.

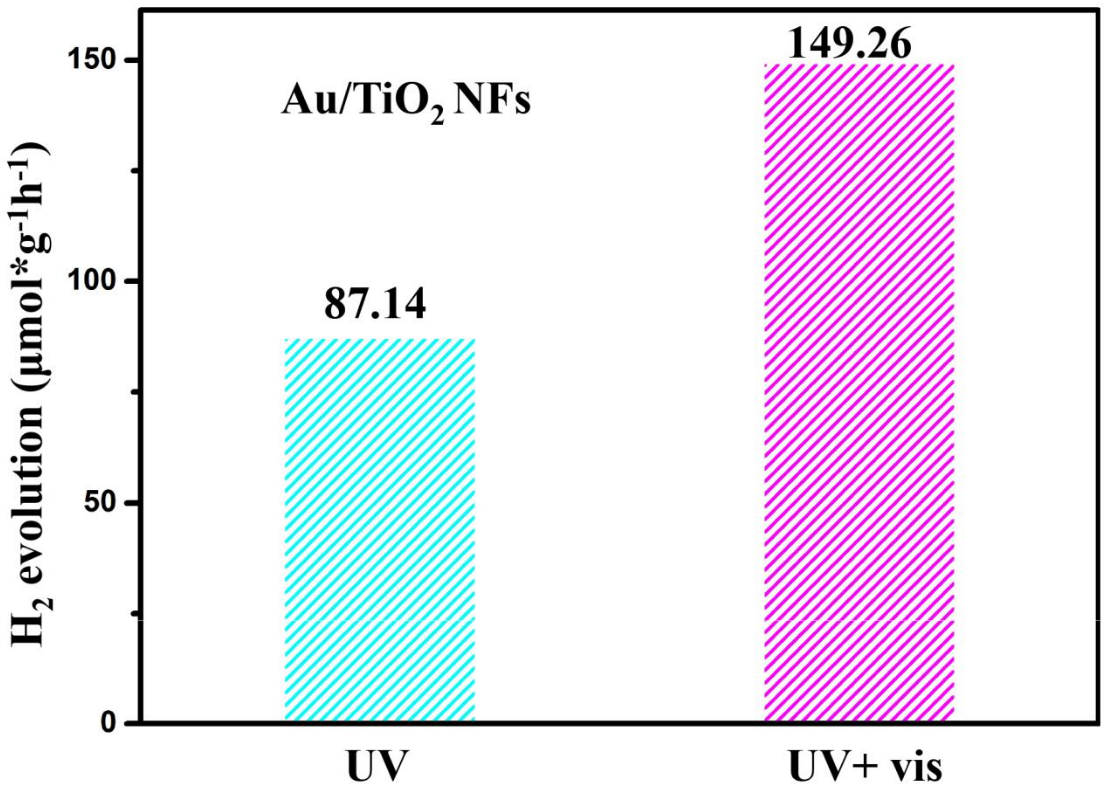

Supplementary Figure S3. Hydrogen production rate of Au/TiO_2 nanofibers under different light irradiation

For the purpose of better verifying the effect of Au SPR of photocatalysts, we prepared samples of Au/TiO_2 NFs and studied its photocatalytic performance, the hydrogen production rate is shown in Figure S3, from the figure we can see that the hydrogen production rate of Au/TiO_2 nanofibers under UV and UV+ vis is 87.14μmol/h and 149.26μmol/h, respectively. The hydrogen production rate under UV+ vis irradiation is much higher than the hydrogen production rate under UV irradiation. This also confirms that the SPR effect of Au promoting the hydrogen production.

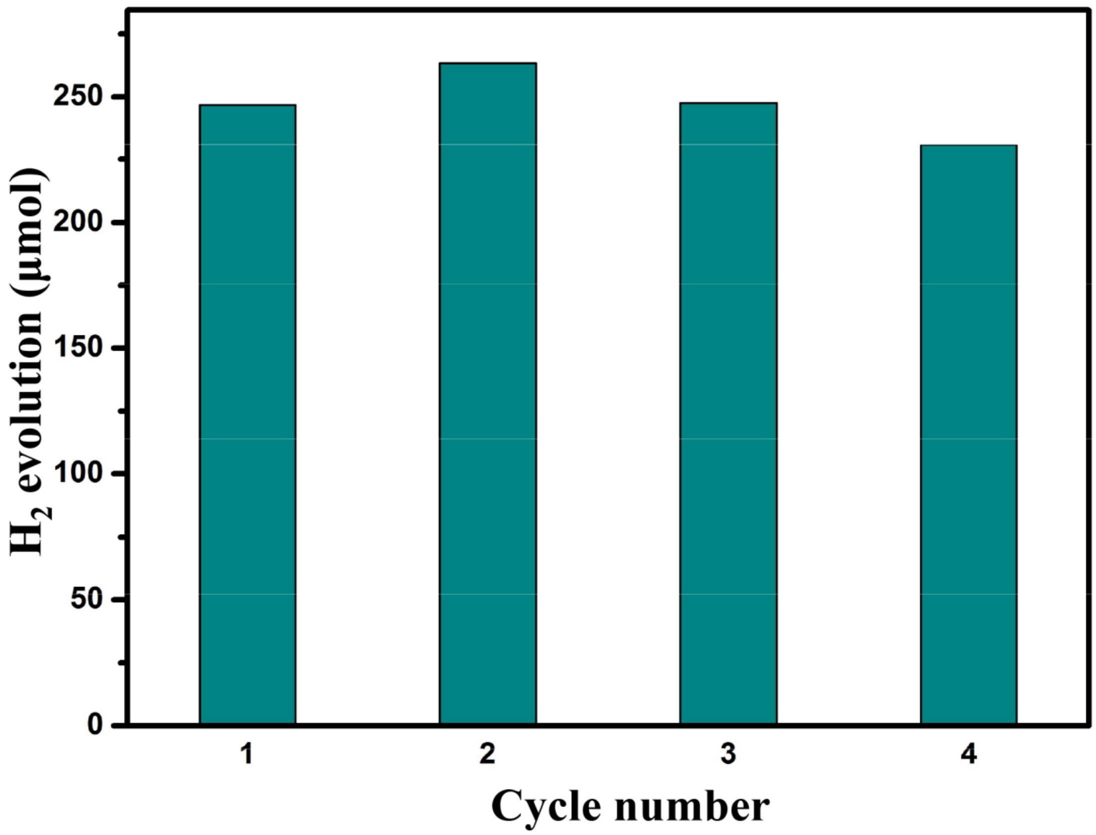

Supplementary Figure S4. Photocatalytic activity of S3 with four times cycling.

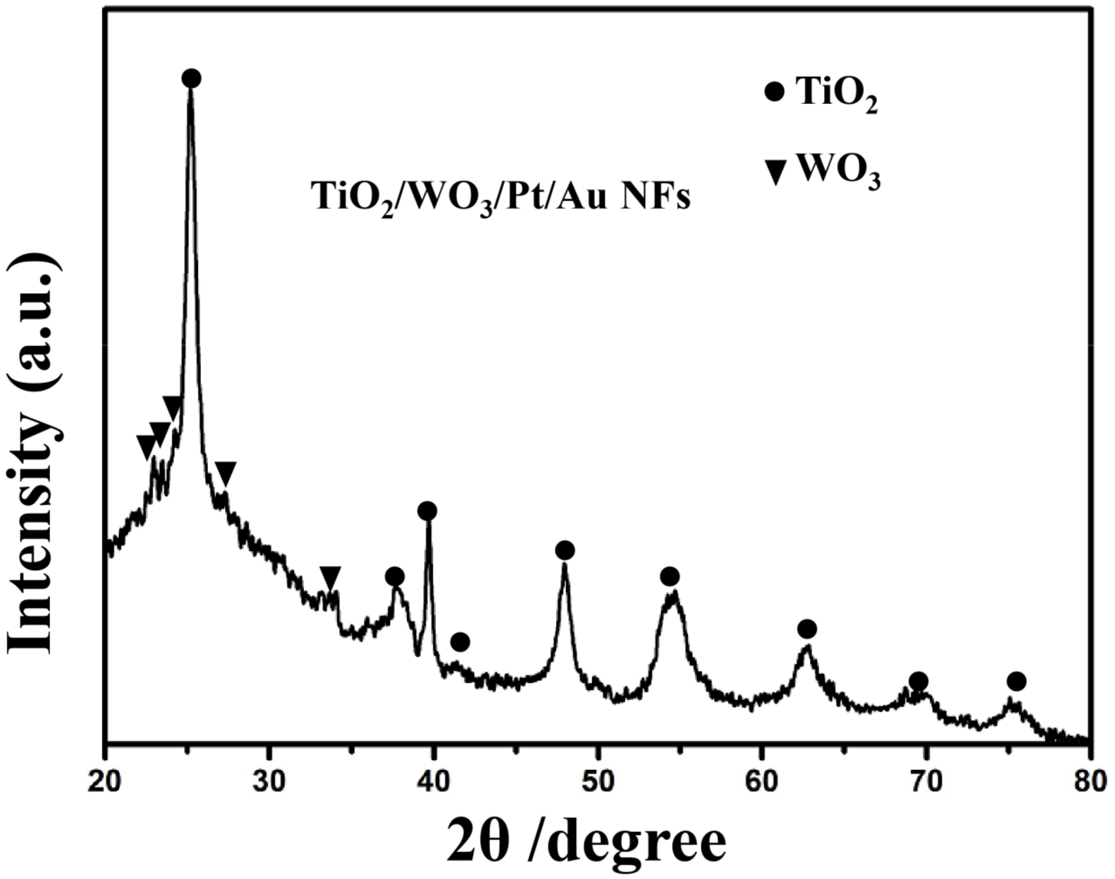

Supplementary Figure S5. XRD pattern of the sample after four times cycling.

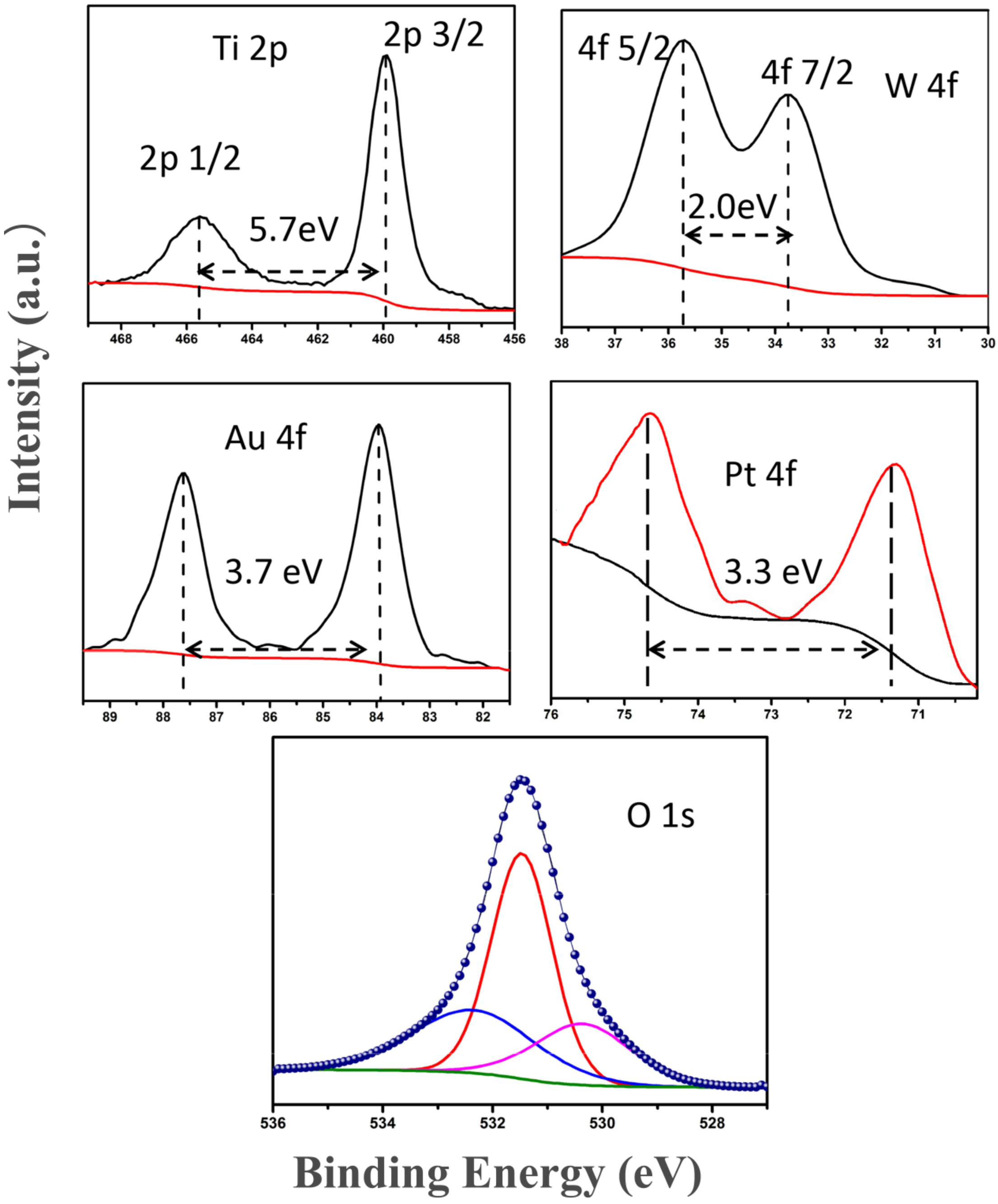

Supplementary Figure S6. XPS of the sample after four times cycling.

Cycling tests were proceeded and shown in Supplementary Figure S4, each experiment was carried out under identical conditions, and the photocatalytic activity of the sample S3 remained a very small change in the four recycling reactions. It is worth mentioning that in this work the samples were one-dimensional nanofiber and

could be easily separated from an aqueous suspension, owing to the large length to diameter ratio. Moreover, the XRD and XPS analysis after catalytic action were added in Supplementary figure S5 and Supplementary figure S6. All the results indicate the excellent stability of photocatalysts.
